# Blood Pressure-Lowering Effects of Finerenone: A Systematic Review and Meta-Analysis of Randomized Controlled Trials

**DOI:** 10.3390/jcm15197694

**Published:** 2026-10-04

**Authors:** Gaetano Marino, Stefania Angela Di Fusco, Elvira Graziano, Sara Corradetti, Stefano Aquilani, Silvio Fedele, Federico Nardi, Domenico Gabrielli, Carmine Savoia, Furio Colivicchi

**Affiliations:** 1 Clinical and Rehabilitation Cardiology Unit, San Filippo Neri Hospital, ASL Roma 1, 00135 Rome, Italy; 2 Independent Researcher, 00189 Rome, Italy; 3 Interventional Cardiology, Sandro Pertini Hospital, 00157 Rome, Italy; 4 Division of Cardiology, Santo Spirito Hospital, 15033 Alessandria, Italy; 5 Department of Cardio-Thoracic and Vascular Medicine and Surgery, Division of Cardiology, S. Camillo-Forlanini Hospital, 00152 Rome, Italy; 6 Clinical and Molecular Medicine Department, Faculty of Medicine and Psychology, Sant’Andrea Hospital, Sapienza University of Rome, 00189 Rome, Italy

**Keywords:** finerenone, blood pressure lowering, mineralocorticoid receptor antagonists

## Abstract

**Background:** Optimal blood pressure (BP) control remains elusive for millions of patients worldwide, often leading to resistant or secondary hypertension. Overactivation of the mineralocorticoid receptor (MR) drives this systemic pathology. While steroidal mineralocorticoid receptor antagonists (MRAs) are effective, their clinical utility is limited by poor cross-selectivity and risk of hyperkalemia. Finerenone, a novel non-steroidal MRA, offers high selectivity and potent target-organ protection, but a comprehensive pooling of its direct impact on BP is still needed. **Methods:** Following the PRISMA guidelines, a systematic search was conducted across PubMed, Embase, and ClinicalTrials.gov up to August 2026 for randomized controlled trials (RCTs) comparing finerenone versus placebo and reporting BP outcomes. The primary outcome was office systolic blood pressure (SBP). **Results:** Six RCTs involving 19,609 patients were included. Finerenone demonstrated a statistically significant but modest reduction in both office SBP (LSM difference: −2.83 mmHg; 95% CI: −3.15 to −2.51; *p* < 0.001) and office diastolic blood pressure (DBP) (LSM difference: −1.04 mmHg; 95% CI: −1.37 to −0.70; *p* < 0.001) compared with the placebo, with an absence of statistical heterogeneity (I^2^ = 0.00%). Subgroup analysis confirmed office SBP-lowering efficacy across different clinical phenotypes, including chronic kidney disease and heart failure (*p* for interaction = 0.18). In a small subset of patients, finerenone reduced 24-h ambulatory SBP (LSM difference: −7.88 mmHg; 95% CI: −10.94 to −4.82; *p* < 0.001), though these exploratory findings warrant caution due to the limited sample size; no significant reduction was observed for 24-h ambulatory DBP. **Conclusions:** Finerenone is associated with a modest but statistically significant reduction in systemic SBP.

## 1. Introduction

Hypertension remains a leading global driver of cardiovascular morbidity, mortality, and chronic kidney disease (CKD) [1]. Despite the widespread availability of conventional antihypertensive regimens, optimal blood pressure (BP) control remains elusive for millions of patients worldwide, frequently culminating in resistant or secondary hypertensive phenotypes [2]. Overactivation of the mineralocorticoid receptor (MR) plays a foundational role in this systemic pathology, driving intravascular volume expansion, endothelial dysfunction, target-organ inflammation, and progressive tissue fibrosis. Consequently, MR antagonism represents a crucial therapeutic strategy in modern cardiorenal medicine [3]. For decades, pharmacological options targeting this pathway were strictly limited to first- and second-generation steroidal mineralocorticoid receptor antagonists (MRAs), such as spironolactone and eplerenone. While clinically effective, the structural scaffold of steroidal MRAs often leads to poor cross-selectivity [4]. Spironolactone exhibits significant off-target binding to progesterone and androgen receptors, frequently causing dose-dependent, treatment-limiting side effects such as gynecomastia, erectile dysfunction, and menstrual irregularities. Conversely, eplerenone is highly selective but features significantly lower antihypertensive potency, alongside substantial cost burdens that restrict its global access. Furthermore, both steroidal agents carry a high propensity for inducing severe hyperkalemia, particularly in patients with pre-existing renal impairment [5]. Finerenone has emerged as a novel, next-generation, non-steroidal MRA characterized by a unique bulky chemical structure and distinct physiochemical properties. Unlike its steroidal predecessors, finerenone binds to the MR with high affinity and exceptional selectivity, acting as an inverse agonist that effectively blocks MR nuclear translocation and inhibits inflammatory and fibrotic gene expression without inducing any sex hormone receptor-related adverse events. Preclinical and clinical evidence has established that finerenone achieves a balanced distribution between cardiac and renal tissues, conferring potent target-organ protection alongside a highly stable potassium safety profile [6]. Given these unique pharmacodynamic benefits, the global clinical application of finerenone has rapidly expanded. Beyond its established regulatory indications for slowing kidney disease progression in type 2 diabetes, there is growing interest in its broader utility for systemic blood pressure management across complex patient spectrums, including resistant essential hypertension, heart failure, and primary aldosteronism [7]. However, a comprehensive pooling of its direct effects on office and ambulatory blood pressure across different trial frameworks is still needed to fully characterize its systemic efficacy. Therefore, this systematic review and meta-analysis aims to evaluate the precise impact of finerenone on both systolic and diastolic blood pressure profiles.

## 2. Methods

### 2.1. Data Source and Search Strategy

This meta-analysis was conducted in accordance with the PRISMA (Preferred Reporting Items for Systematic Reviews and Meta-Analyses) guidelines [8]. The meta-analysis was registered on the International Prospective Register of Systematic Reviews (PROSPERO; registration no. CRD420261488194. A systematic search was conducted across PubMed, Embase and ClinicalTrials.gov from inception to August 2026. The following search term was used: “finerenone”. The search strategy and PRISMA checklist are available in the Supplementary Materials.

### 2.2. Trial Selection and Eligibility Criteria

The PICOS methodology (Population, Intervention, Comparison, Outcomes, Study design) was used for trial selection [9]. We included only RCTs that met the following criteria:

- Study population consisted of adults aged ≥18 years. Eligible studies were included irrespective of the participants’ cardiovascular health status at baseline;
- RCTs comparing finerenone versus placebo;
- Trials reporting variations in blood pressure levels as outcomes.

The exclusion criteria were:

- Studies including interventions other than finerenone;
- Observational studies, case reports, reviews, non-randomized studies, studies with insufficient data for analysis and non-human clinical trials;
- There were no language restrictions and trials of any sample size, follow-up duration and from any country were included.

Office systolic blood pressure (SBP) was the primary outcome of this study.

Office diastolic blood pressure (DBP), 24-h ambulatory blood pressure monitoring systolic (ABPM SBP), and 24-h ambulatory blood pressure monitoring diastolic (ABPM DBP) were identified as secondary outcomes.

Two independent reviewers (G. M. and E. G.) examined and selected the eligible studies. This process involved two phases: in the first screening phase, search results were considered based on the title and abstract; in the second phase, studies were evaluated by examining the full text and excluding those with insufficient data and full text unavailable. Disagreements were resolved by discussion and consensus. Using an Excel spreadsheet, the following data regarding the study characteristics were extracted: first author, year of publication, name of the trial, study design, study duration, finerenone doses, number of patients and concomitant antihypertensive therapy. During the preparation of this manuscript, the authors used Gemini (Google) for the purpose of improving the readability and linguistic quality of the text. The authors have reviewed and edited the output and take full responsibility for the content of this publication.

### 2.3. Statistical Analysis

For each study included in the meta-analysis, effect measures and the 95% confidence interval (CI) were calculated by the inverse-variance method. The random-effects REstricted Maximum Likelihood (REML) statistical model was applied to synthesize the effect sizes of the studies assuming interstudy heterogeneity. For our outcomes, least squares mean (LSM) difference with 95% CI was calculated. The findings were used to generate forest plots. The presence of publication bias was investigated through funnel plots, Egger’s test, Begg’s test and trim, and fill analysis. Heterogeneity across studies was assessed using the “τ^2^” and the “Higgins and Thompson’s I^2^”. Heterogeneity was considered to be low, moderate, or high if I^2^ was 25%, 50%, or 75%, respectively [10,11]. Sensitivity analysis using the leave-one-out method was performed to identify any outliers. Subgroup analysis of the outcome was performed to compare finerenone versus the placebo across specific patient categories. A random-effects meta-regression was conducted to explore potential sources of heterogeneity for the outcome, considering covariates such as year of the study and duration of the study. The regression coefficients and 95% CI were calculated for each covariate; bubble plots were generated to visualize the relationship between the analyzed covariates and the treatment effect size. Statistical significance was set at *p* < 0.05 (2-tailed). Statistical analysis was performed using StataSE 19.

### 2.4. Certainty of Evidence

The GRADE (Grading of Recommendations Assessment, Development and Evaluation) method was used to assess the quality and strength of the evidence [12]. GRADE uses a structured approach to assign a rating of high, moderate, low, or very low certainty to the evidence. The quality of evidence according to the GRADE method considers several factors such as study design, risk of bias, inconsistency, indirectness, imprecision, and publication bias. Two different reviewers rated the quality of the evidence for each outcome independently. The GRADEpro GDT (Guideline Development Tool) software was used to generate an evidence profile table (Supplementary Materials).

## 3. Results

**Figure 1:**
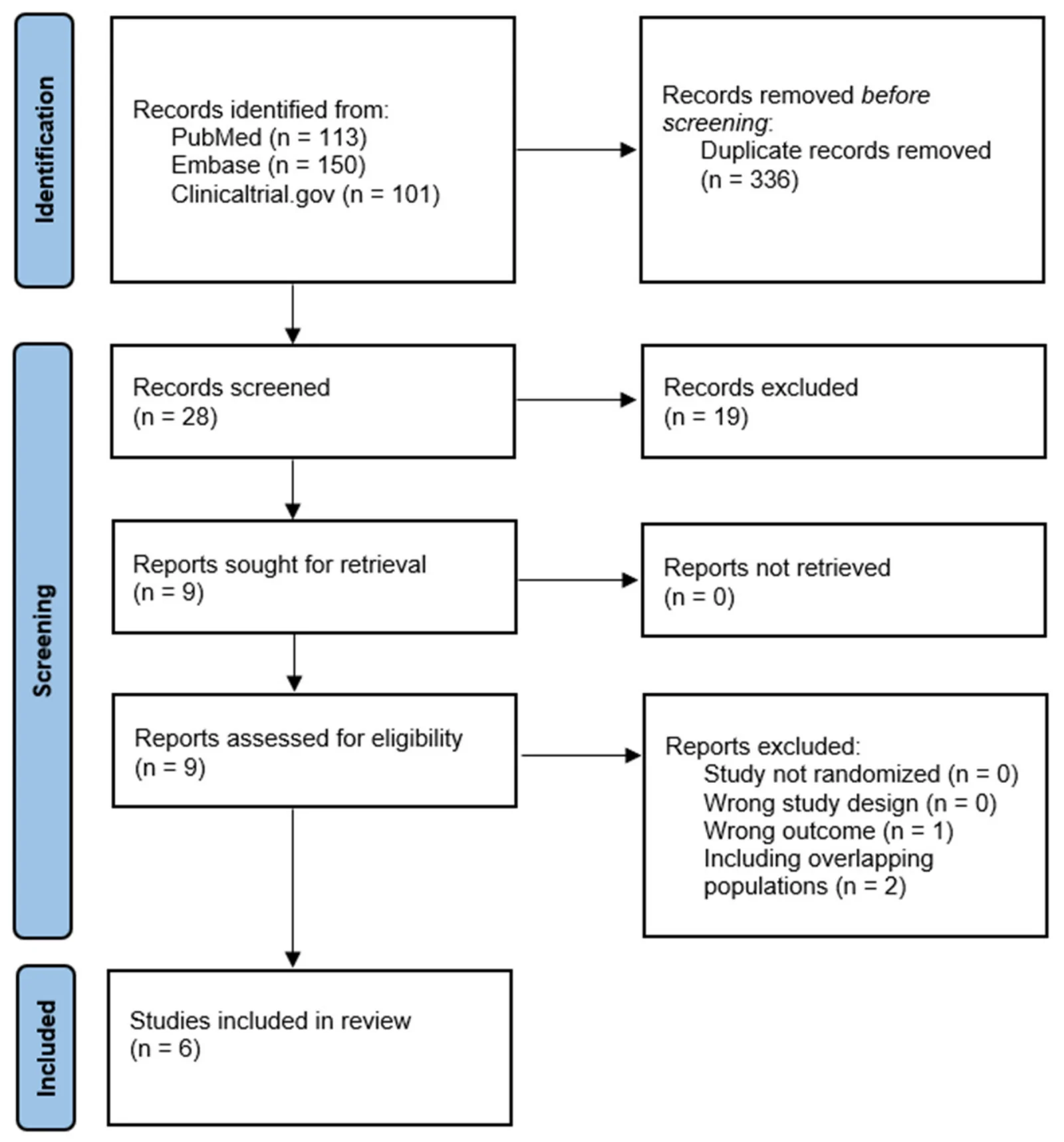
Study selection flow diagram.

We identified 364 articles. A total of 336 were removed as duplicates. In the first screening phase, 28 papers were examined on the basis of the title and abstract contents. Of these, 19 articles were excluded because they did not fulfill the inclusion criteria. In a second phase, through full-text evaluation of the 9 articles eligible for inclusion, 1 was excluded for wrong outcome, and 2 for overlapping population. At the end of this process, 6 clinical trials were included for the purpose of this systematic review and meta-analysis [13,14,15,16,17,18]. A search flow diagram illustrating the study selection process is shown in Figure 1.

The entire population recruited consisted of 19,609 patients (9905 in the finerenone group and 9704 in the placebo group). The follow-up duration varied between 3 and 32 months. The characteristics of the included trials and the patient populations are presented in Table 1.

### 3.1. Risk of Bias and Quality Assessment

The quality of the included studies was assessed using the Cochrane Risk of Bias tool for randomized controlled trials. The Cochrane Risk of Bias tool is structured into seven different domains for potential sources of bias, which were examined by a pair of independent reviewers. The different domains identified and evaluated are illustrated in Table 2.

To mitigate the risk of reporting bias and ensure methodological transparency, the review was conducted in strict adherence to the PRISMA 2020 guidelines. To minimize subjectivity, each stage of data selection and extraction was performed in a double-blind manner by two independent reviewers. The systematic assessment of the included RCTs revealed an overall low risk of bias across the evaluated domains. For random sequence generation (D1) and allocation concealment (D2), all trials provided detailed methodologies confirming appropriate randomization frameworks. Performance bias (D3) and detection bias (D4) were minimized through robust double-blind designs involving both participants and investigators. Furthermore, attrition bias (D5) and reporting bias (D6) were categorized as low risk due to complete outcome reporting and predefined trial protocols, guaranteeing methodological transparency.

The certainty of evidence for the evaluated outcomes was systematically assessed using the GRADE framework. For the primary outcome of office systolic blood pressure levels, the body of evidence from the six randomized controlled trials was graded as high. This rating was supported by the absence of serious concerns regarding the risk of bias, inconsistency, indirectness, or imprecision across the included literature. The complete GRADE evidence profile table is detailed in the Supplementary Materials.

#### Office SBP

**Figure 2:**
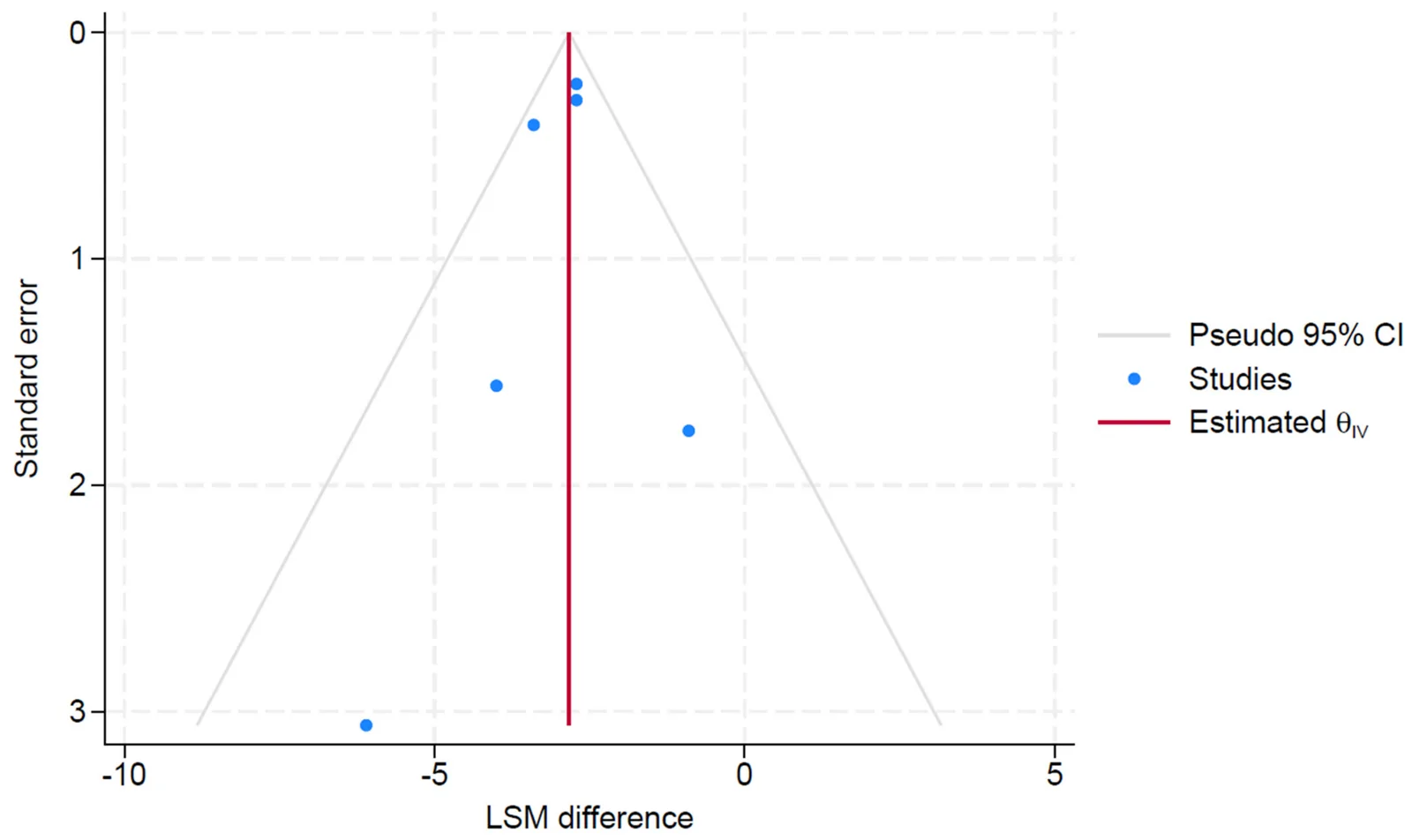
Funnel plots assessing publication bias. The y-axis represents study precision (standard error) while the x-axis shows LSM difference.

For our primary outcome, statistical tests for publication bias (Egger’s test and Begg’s test), funnel plot (Figure 2) and trim and fill analysis assessing potential publication bias revealed no significant asymmetries. Specifically the Egger’s test provided no evidence of publication bias for our outcome (*p* = 0.43). Begg’s rank correlation test revealed no statistically significant asymmetry among the included studies (Kendall’s score = −3.00, Z = −0.75, *p* = 0.71), indicating no evidence of small-study effects. To further evaluate the robustness of the pooled findings, a non-parametric trim-and-fill sensitivity analysis was conducted using a random-effects model with the REML method. The algorithm identified one potentially missing study on the right side of the funnel plot to achieve symmetry. However, the imputation of this missing study did not meaningfully alter the overall effect size. The pooled LSM difference shifted negligibly from the observed estimate of −2.83 mmHg (95% CI: −3.15 to −2.51) to a corrected estimate of −2.82 (95% CI: −3.14 to −2.50).

**Figure 3:**
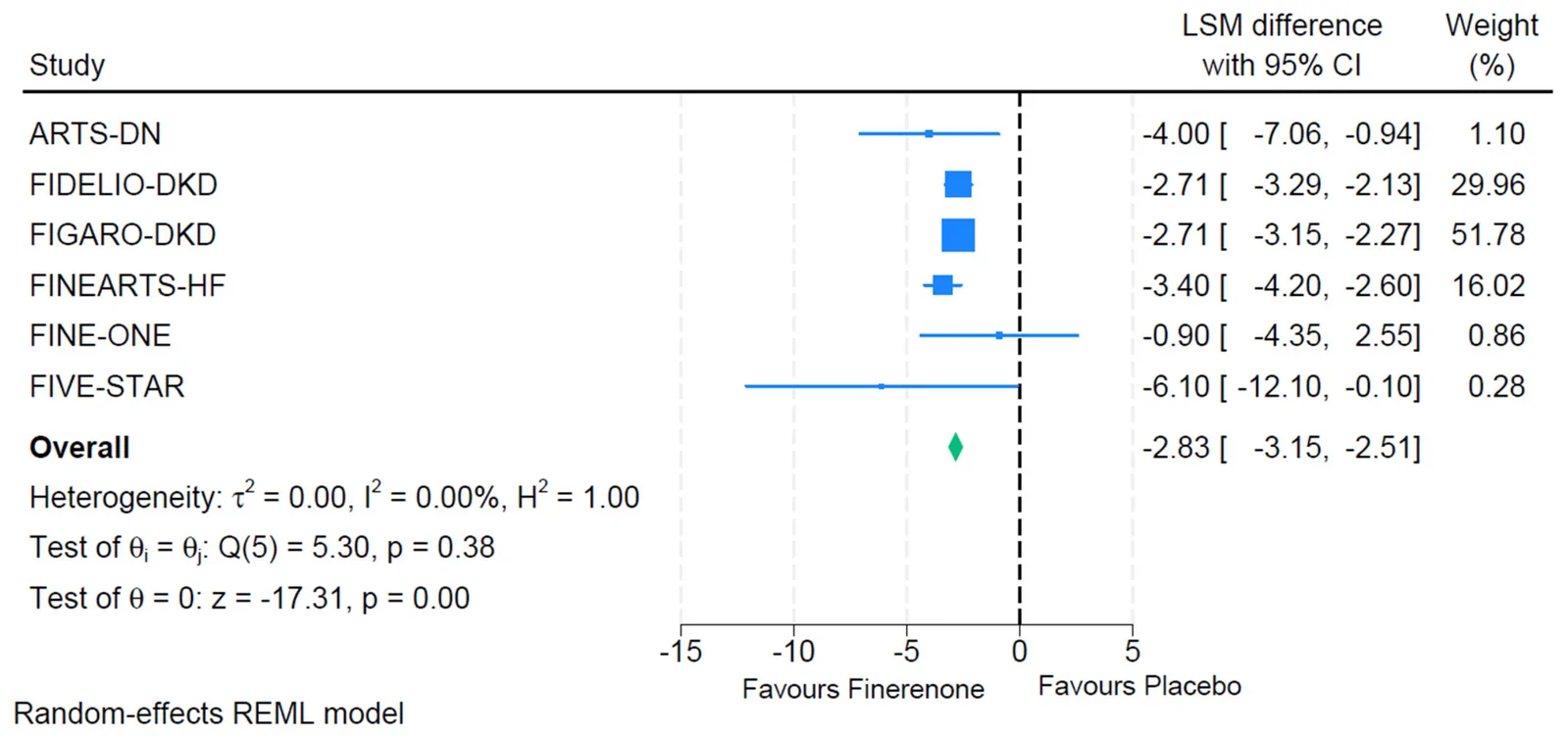
Forest plot assessing the effect of finerenone on office SBP.

A pooled meta-analysis was conducted to evaluate the efficacy of finerenone compared with the placebo in reducing office SBP across six trials (ARTS-DN, FIDELIO-DKD, FIGARO-DKD, FINEARTS-HF, FINE-ONE, and FIVE-STAR). The overall pooled analysis demonstrated a statistically significant reduction in office SBP favoring finerenone over the placebo, with an overall LSM difference of −2.83 mmHg (95% CI: −3.15, −2.51; *p* < 0.001) (Figure 3). Among the included studies, the largest statistical weights were attributed to the FIGARO-DKD trial (51.78% weight; LSM difference: −2.71 mmHg; 95% CI: −3.15, −2.27) and the FIDELIO-DKD trial (29.96% weight; LSM difference: −2.71 mmHg; 95% CI: −3.29, −2.13), followed by the FINEARTS-HF trial (16.02% weight; LSM difference: −3.40 mmHg; 95% CI: −4.20, −2.60). While smaller Phase II dose-finding or specialized trials exhibited wider confidence intervals due to lower statistical weights, a trend toward office SBP reduction was observed across all sub-analyses. The corrected pooled estimate for the ARTS-DN trial yielded an LSM difference of −4.00 mmHg (95% CI: −7.06, −0.94 mmHg; weight: 1.10%). The remaining small-scale trials, FINE-ONE and FIVE-STAR, yielded LSM differences of −0.90 mmHg (95% CI: −4.35, 2.55 mmHg) and −6.10 mmHg (95% CI: −12.10, −0.10 mmHg), respectively. Assessment of heterogeneity using the random-effects REML model revealed negligible inconsistency across the trials, with an I^2^ value of 0.00% (τ^2^ = 0.00, H^2^ = 1.00). However, this statistical finding should not obscure the presence of clinical diversity across the included trials regarding patient populations, baseline characteristics, and dosing regimens. The test for study differences was not statistically significant (*p* = 0.38), indicating a uniform treatment effect of finerenone on office SBP across the evaluated cohorts. The overall effect test confirmed high statistical significance (*p* = 0.00).

**Figure 4:**
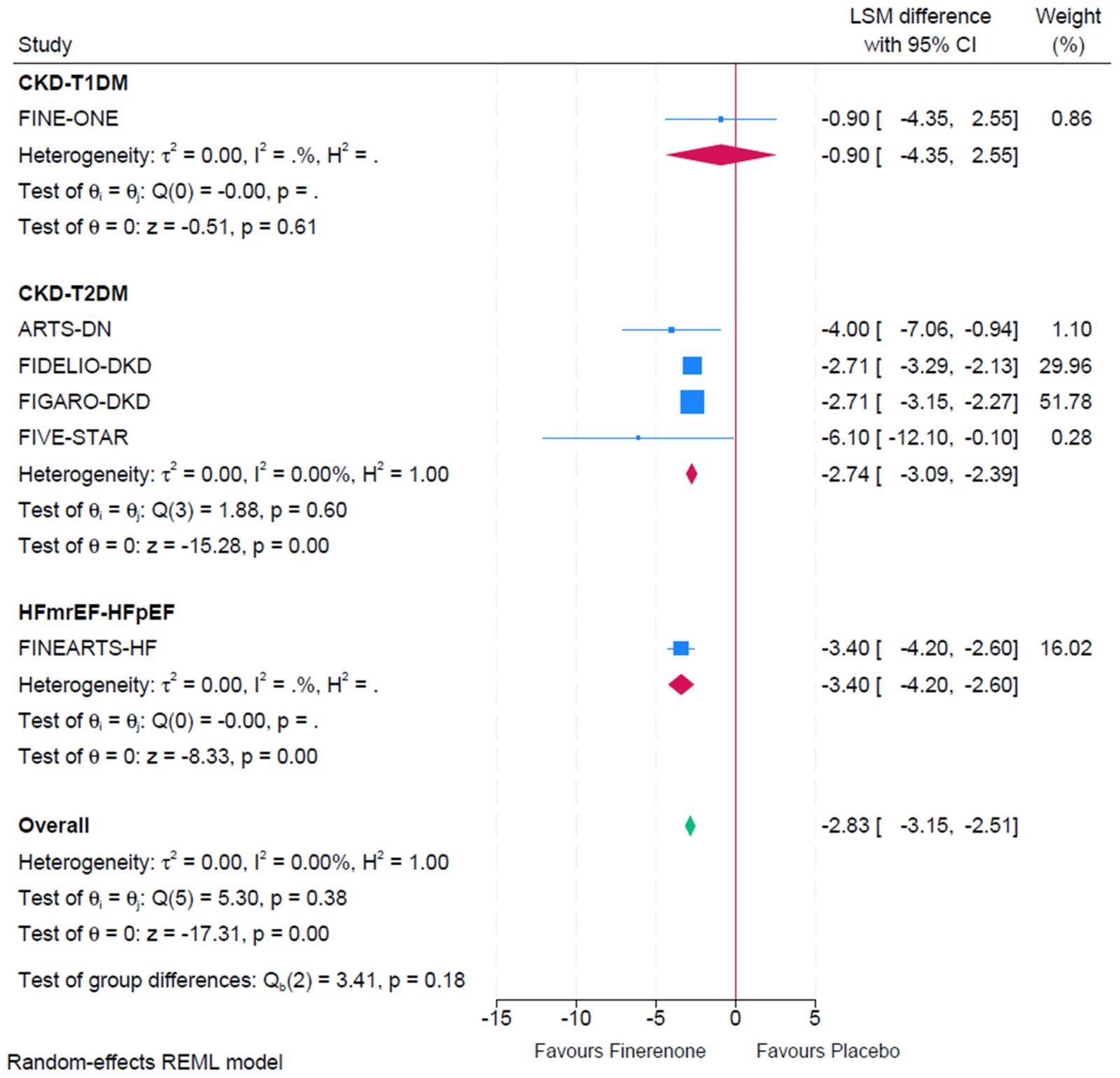
Subgroup analysis forest plot evaluating the blood pressure-lowering effect of finerenone on office SBP across different classes.

To evaluate whether the systemic blood pressure-lowering effect of finerenone varied across different patient populations, a subgroup analysis was performed based on three clinical phenotypes: chronic kidney disease in type 1 diabetes mellitus (CKD-T1DM), chronic kidney disease in type 2 diabetes mellitus (CKD-T2DM), and heart failure with mildly reduced or preserved ejection fraction (HFmrEF-HFpEF) (Figure 4).

In patients with CKD-T2DM, the largest subgroup comprising four trials (ARTS-DN, FIDELIO-DKD, FIGARO-DKD, and FIVE-STAR), finerenone induced a statistically significant reduction in office SBP, with a pooled LSM difference of −2.74 mmHg (95% CI: −3.09, −2.39 mmHg; *p* < 0.001). Within-group heterogeneity for this subset was absent (τ^2^ = 0.00, I^2^ = 0.00%, Q = 1.88, *p* = 0.60).

Similarly, in the HFmrEF-HFpEF population (represented by the FINEARTS-HF trial), a reduction in office SBP was observed, yielding an LSM difference of −3.40 mmHg (95% CI: −4.20, −2.60 mmHg; *p* < 0.001). Conversely, in the CKD-T1DM subgroup (represented by the single FINE-ONE trial), the reduction in office SBP did not reach statistical significance, with an LSM difference of −0.90 mmHg (95% CI: −4.35, 2.55 mmHg; *p* = 0.61).

Crucially, the formal test for subgroup interactions revealed no statistically significant differences in the blood pressure response among the three clinical categories (*p* = 0.18). This statistical homogeneity indicates that the modest systemic blood pressure-lowering effect of finerenone remains uniform.

### 3.2. Meta-Regression

**Figure 5:**
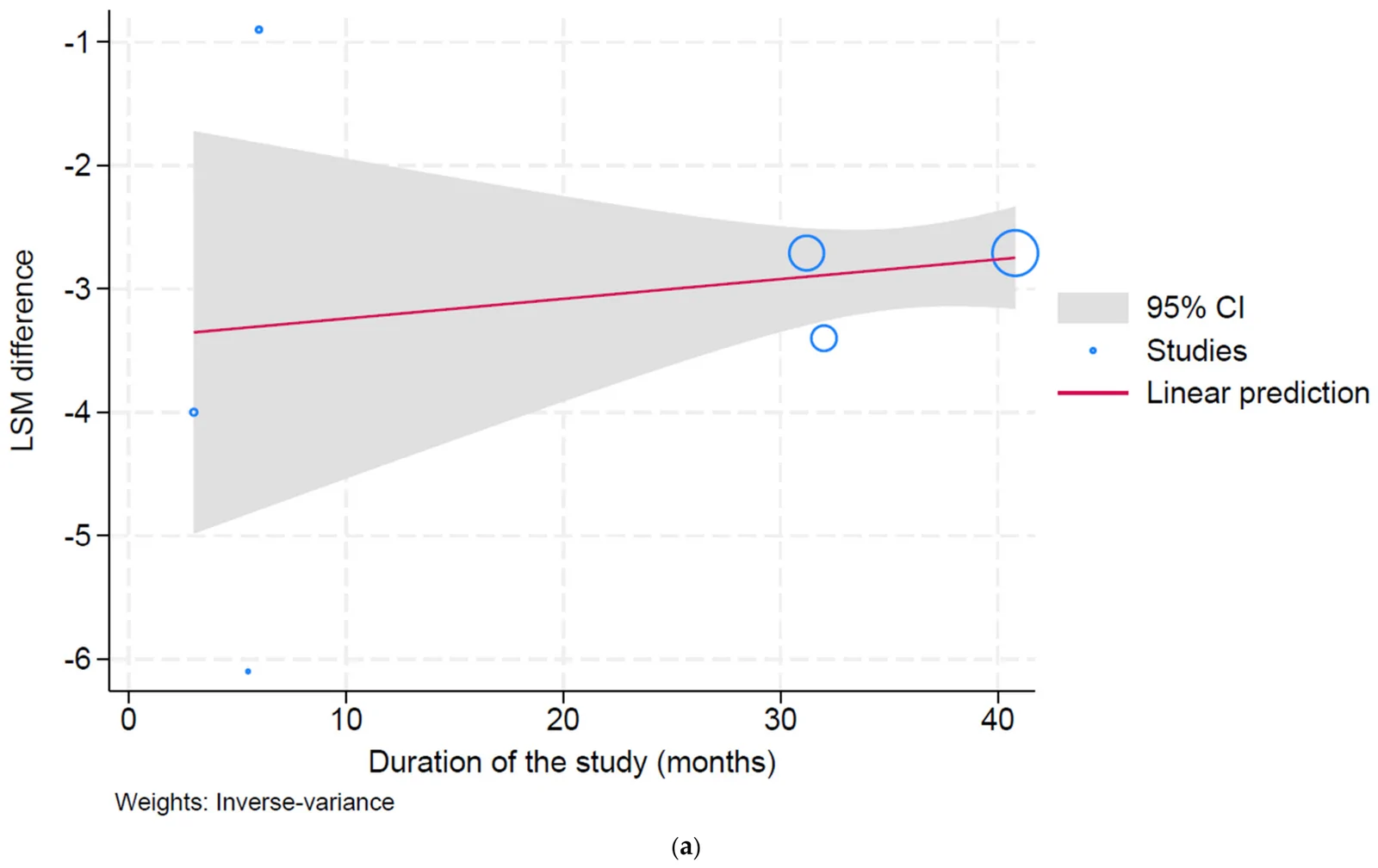

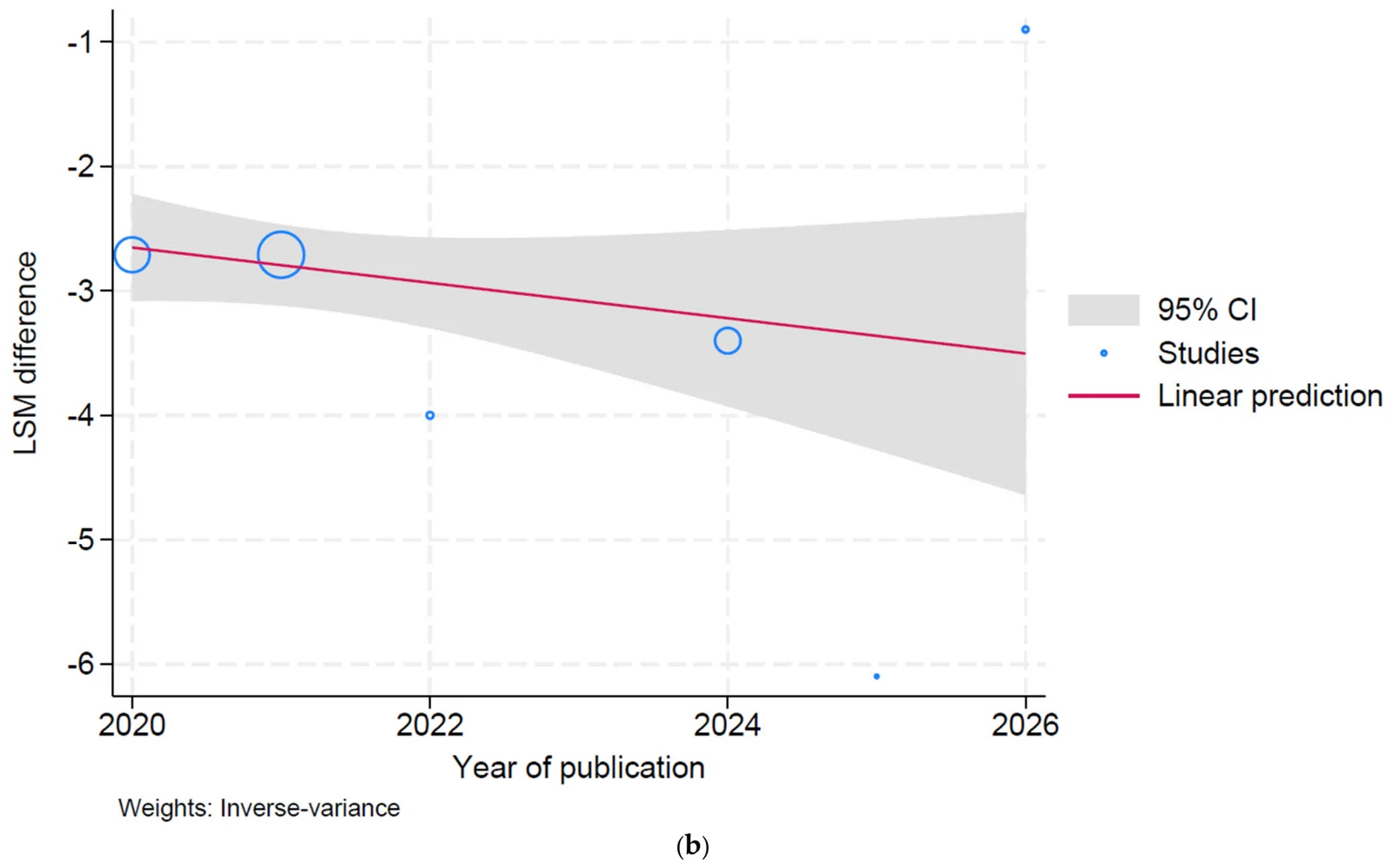
Bubble plot assessing the impact of duration of the study (**a**) and year of publication (**b**) on blood pressure-lowering effect of finerenone on office SBP.

Although the overall analysis demonstrated the absence of statistical evidence of heterogeneity (I^2^ = 0.00%), a series of random-effects meta-regression models was systematically performed to investigate potential sources of study-level heterogeneity and assess whether the antihypertensive effect of finerenone on office SBP was influenced by temporal or trial-design factors. Two separate random-effects meta-regression models were fitted using the restricted maximum likelihood (REML) method. The examined covariates included the overall study duration and the year of publication. The meta-regression evaluating trial duration (months) included data from 6 observations. The analysis revealed no statistically significant association between the duration of the study and the reduction in office SBP, as indicated by a regression coefficient of 0.016 mmHg per month (95% CI: −0.033 to 0.065; *p* = 0.522). The model accounted for 0.00% of the between-study variance (R^2^ = 0.00%), and the Wald χ^2^ test confirmed the lack of overall model significance (Wald χ^2^ = 0.41). Residual heterogeneity remained negligible (I^2^ = 0.89%, τ^2^ = 0.0023), and the test of residual homogeneity was not significant (*p* = 0.300). Similarly, the meta-regression utilizing publication year as a continuous covariate showed no significant linear relationship with the treatment effect size. The meta-regression assessing the impact of the publication year revealed no statistically significant association with the pooled LSM difference in office SBP (coefficient: −0.1419; 95% CI: −0.3711, 0.0872; z = −1.21, *p* = 0.225). The proportion of residual heterogeneity explained by this covariate was null (R^2^ = 0.00%), with an ongoing absence of statistical residual heterogeneity (I^2^ = 0.00%, *p* = 0.431). Neither the chronological timing of the trials nor the length of patient follow-up significantly modified the blood pressure-lowering effect of finerenone (Figure 5a,b).

To evaluate the stability of the primary pooled estimate and to ensure that the findings were not disproportionately driven by any single investigation, a leave-one-out sensitivity analysis was performed by sequentially omitting one trial at a time and recalculating the overall effect.

The execution of this sensitivity analysis confirmed the stability of the primary findings. The pooled LSM difference in office SBP remained remarkably stable across all iterations, ranging from a minimum reduction of −2.72 mmHg (95% CI: −3.07, −2.37 mmHg) when omitting the FINEARTS-HF trial, to a maximum reduction of −3.00 mmHg (95% CI: −3.61, −2.39 mmHg) when omitting the FIGARO-DKD trial.

Importantly, the statistical significance of the treatment effect was uniformly maintained in every analysis iteration, with all configurations yielding a *p*-value < 0.001. The exclusion of the larger-scale trials (FIDELIO-DKD and FIGARO-DKD) predictably resulted in a slight widening of the 95% confidence intervals due to the reduction in total sample size, yet the direction and clinical magnitude of the pooled estimate experienced no significant deviations.

**Figure 6:**
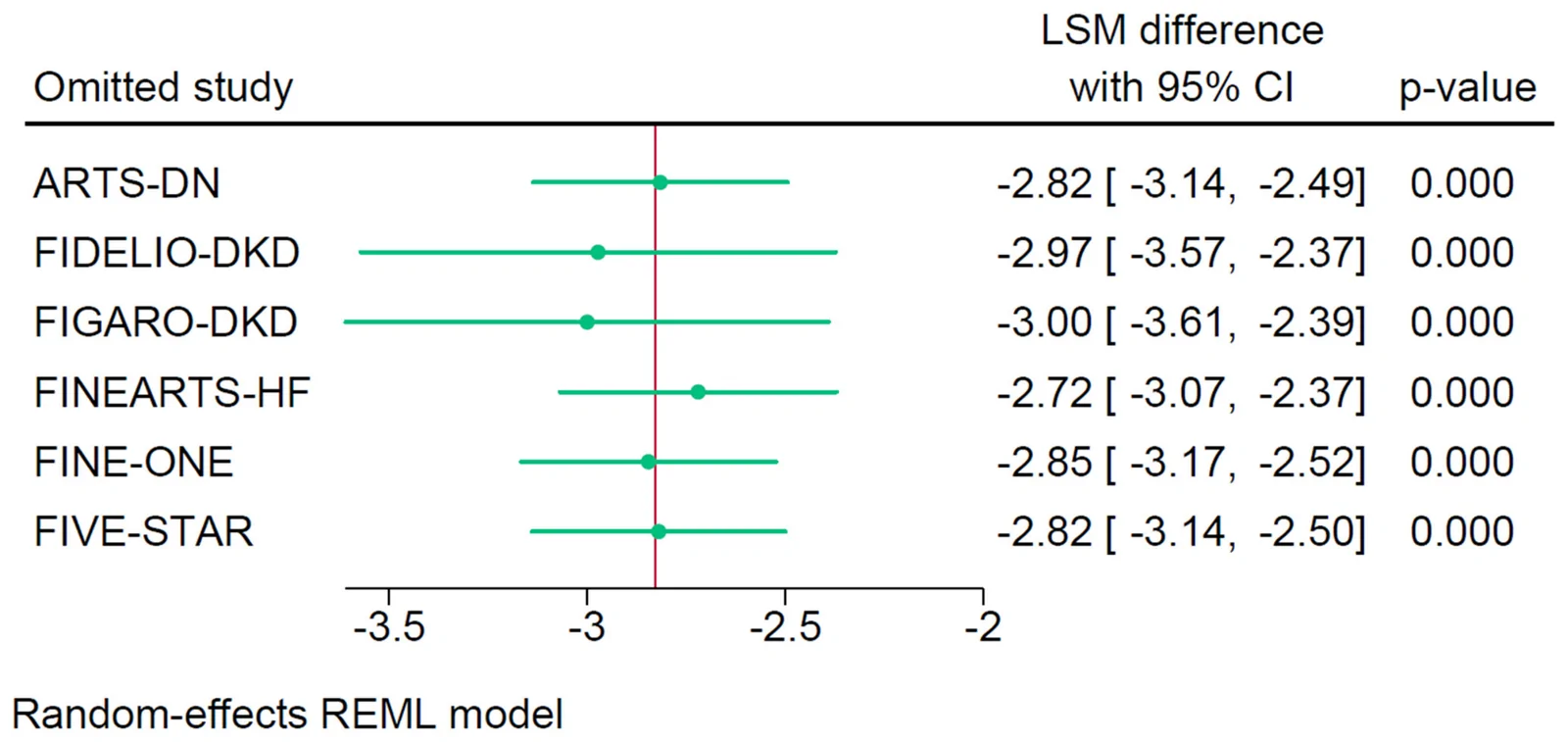
Leave-one-out analysis showing the influence of individual studies on the pooled LSM difference.

These results collectively demonstrate that the overall findings are methodologically sound and not reliant on the presence of any individual outlying study, confirming a stable systemic blood pressure-lowering profile for finerenone (Figure 6).

#### 3.2.1. Office DBP

**Figure 7:**
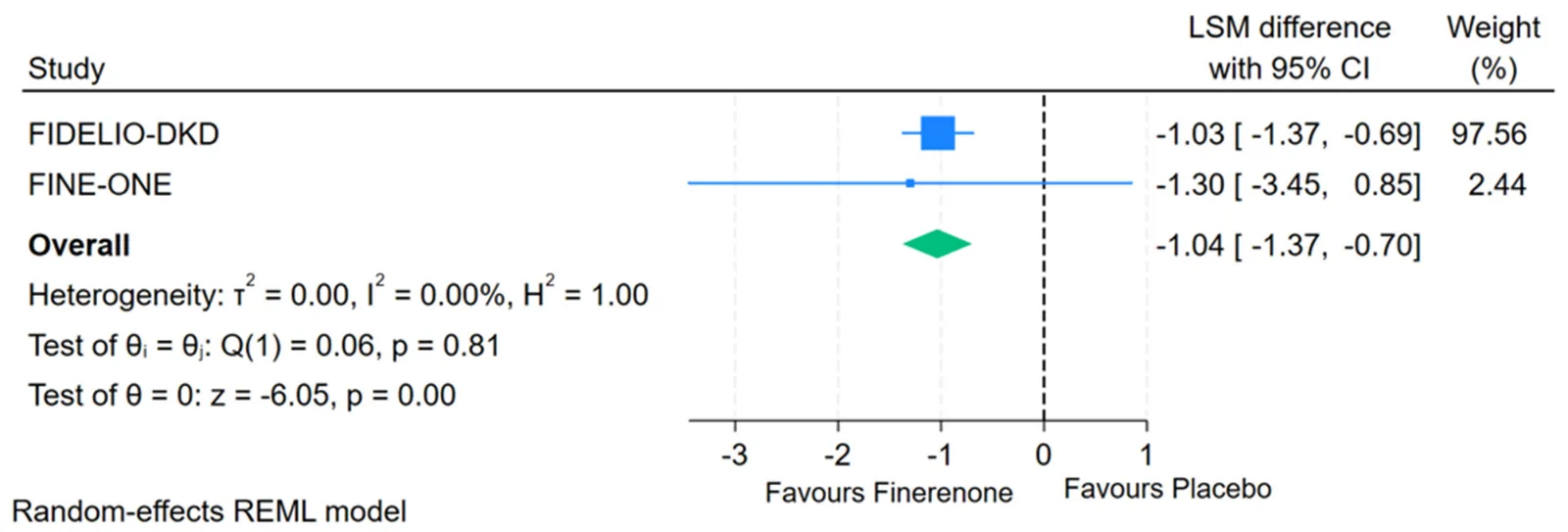
Forest plot assessing the effect of finerenone on office DBP.

A pooled meta-analysis was performed to evaluate the change in office DBP with finerenone compared to the placebo, incorporating data from the FIDELIO-DKD and FINE-ONE trials. The overall pooled analysis demonstrated a statistically significant reduction in office DBP favoring finerenone over the placebo, yielding a pooled LSM difference of −1.04 mmHg (95% CI: −1.37, −0.70; *p* < 0.001). The statistical weight of this analysis was almost entirely driven by the FIDELIO-DKD trial, which accounted for 97.56% of the total weight and reached statistical significance individually, with an LSM difference of −1.03 mmHg (95% CI: −1.37, −0.69). Conversely, the FINE-ONE trial carried a marginal statistical weight (2.44%) and did not reach statistical significance independently, reporting an LSM difference of −1.30 mmHg with a wider confidence interval that crossed the line of no effect (95% CI: −3.45, 0.85). Assessment of heterogeneity using the random-effects REML model demonstrated perfect consistency between the trials, with an I^2^ = 0.00% and τ^2^ = 0.00. The test for study differences confirmed the absence of any significant discrepancy between the two trial estimates (*p* = 0.81). The overall treatment effect test remained significant (*p* = 0.00) (Figure 7).

#### 3.2.2. ABPM SBP

**Figure 8:**
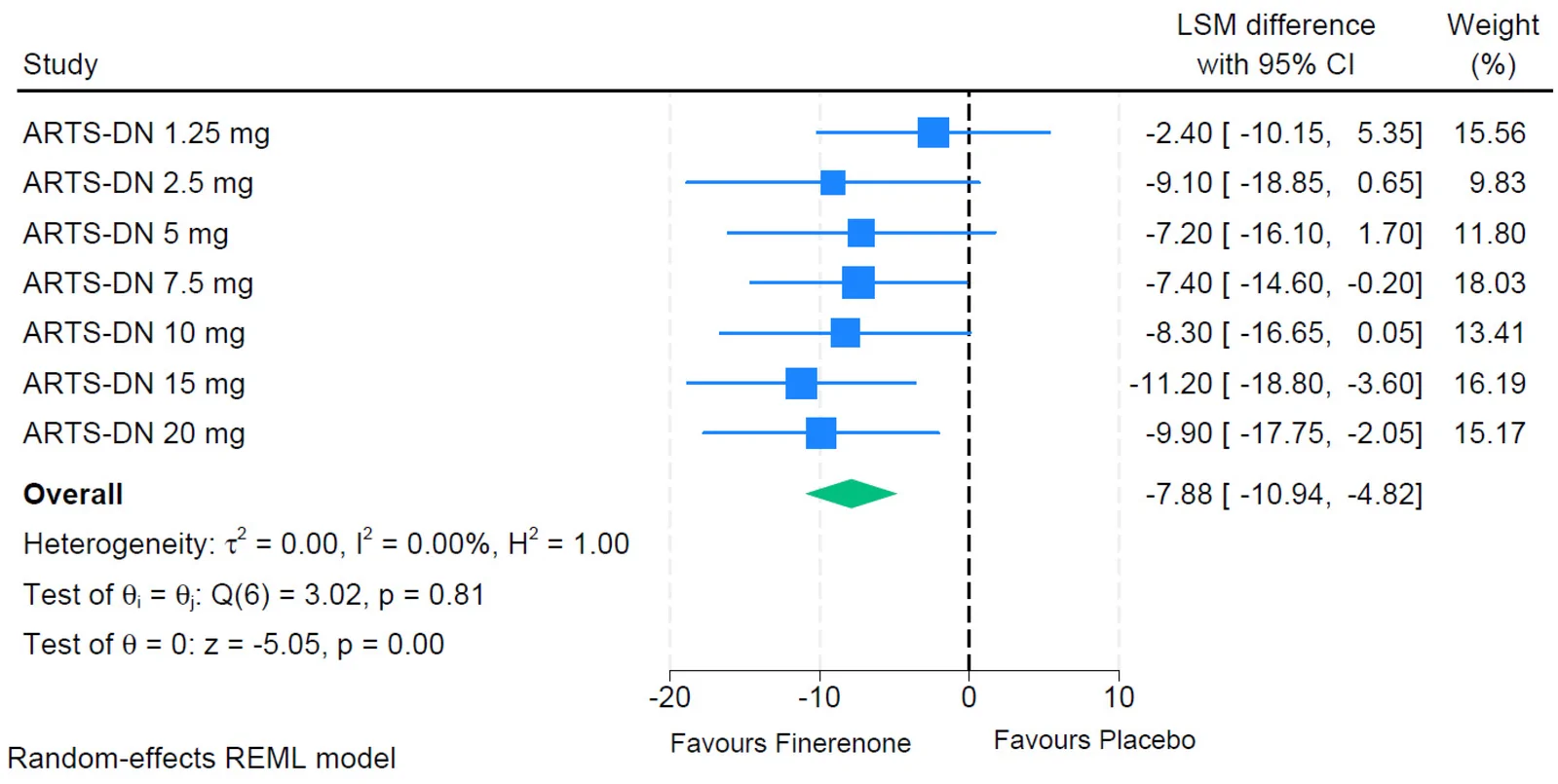
Forest plot assessing the effect of finerenone on ABPM SBP.

To determine the dose-dependent impact of finerenone on ABPM SBP, a pooled analysis was performed across seven distinct dosage arms from the ARTS-DN trial, ranging from 1.25 mg to 20 mg daily. The overall pooled analysis demonstrated a statistically significant reduction in ABPM SBP favoring finerenone over the placebo, with a pooled LSM difference of −7.88 mmHg (95% CI: −10.94, −4.82; *p* < 0.001) (Figure 8). The statistical weights were relatively evenly distributed across the dosage groups, ranging from 9.83% to 18.03%. A descriptive dose–response trend was observed as the daily dose escalated. The lowest dose (1.25 mg) exerted a minimal change in ABPM SBP (LSM difference: −2.40 mmHg; 95% CI: −10.15, 5.35). Moderate reductions were observed with the 2.5 mg (−9.10 mmHg; 95% CI: −18.85, 0.65) and 5 mg arms (−7.20 mmHg; 95% CI: −16.10, 1.70), though neither reached individual statistical significance. The 7.5 mg arm (LSM difference: −7.40 mmHg; 95% CI: −14.60, −0.20) and the 10 mg arm (−8.30 mmHg; 95% CI: −16.65, 0.05) showed more pronounced reductions. The statistically significant reductions in ABPM SBP were achieved in the highest dosage cohorts, specifically the 15 mg arm (LSM difference: −11.20 mmHg; 95% CI: −18.80, −3.60) and the 20 mg arm (LSM difference: −9.90 mmHg; 95% CI: −17.75, −2.05).

**Figure 9:**
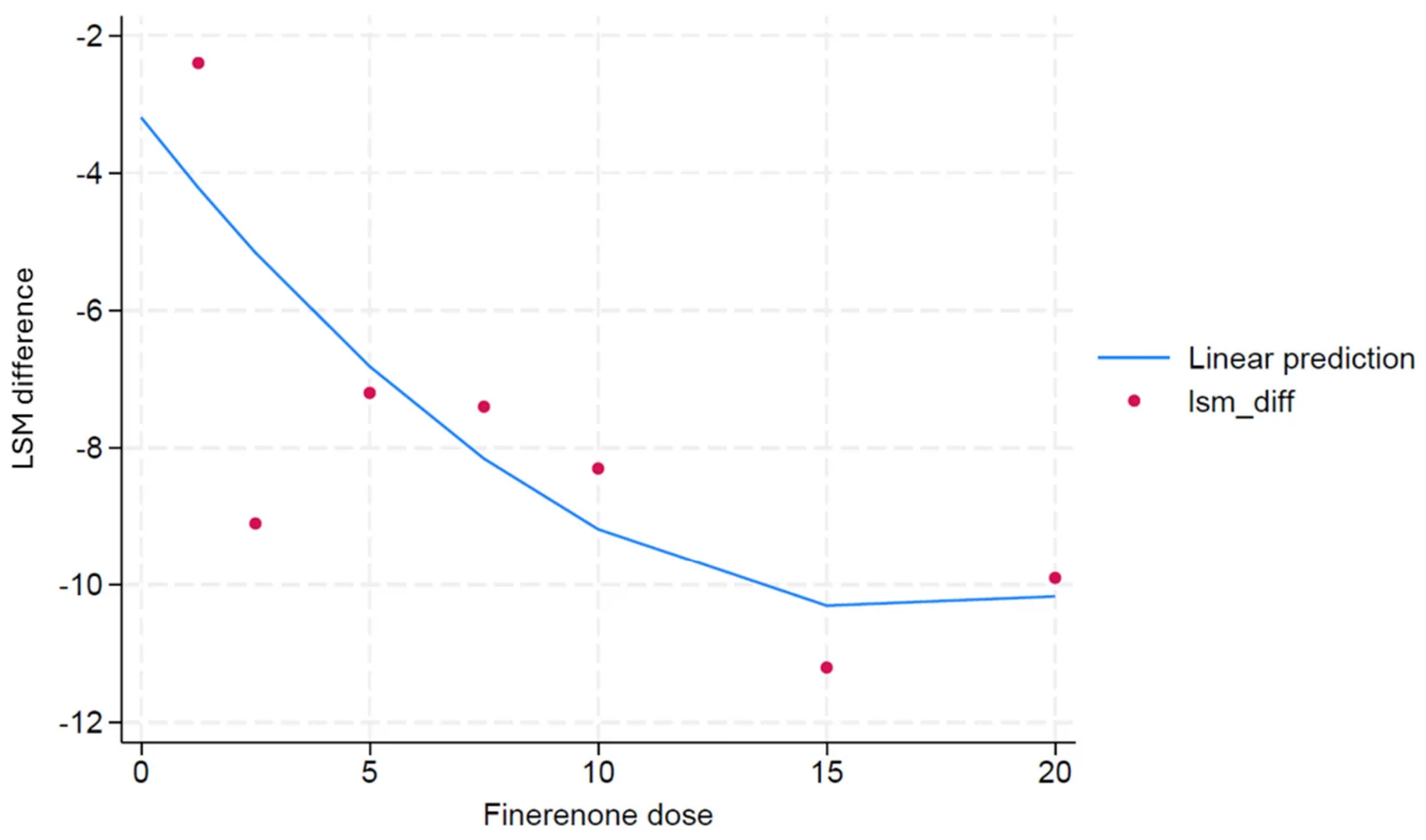
Dose–response effect of finerenone on ABPM SBP.

To explore a non-linear relation, a polynomial random-effects meta-regression model was fitted. The model estimated a linear coefficient of −0.849 mmHg per mg increase (95% CI: −2.754 to 1.055; *p* = 0.382) and a quadratic coefficient of 0.025 mmHg (95% CI: −0.063 to 0.113; *p* = 0.575). Neither the individual parameters nor the omnibus Wald test reached statistical significance (χ^2^ = 2.03, *p* = 0.363, R^2^ = 0.00%). However, visual inspection of the meta-regression curve provides only an exploratory indication of a potential dose-dependent trend, which is heavily characterized by substantial statistical uncertainty at the higher dosage spectrum. The non-linear prediction trend suggests a numerical intensification of the blood pressure-lowering effect from lower doses up to 15 mg; beyond this threshold, the curve visually flattens toward the 20 mg dose. Due to the wide confidence intervals and the limited sample size of the ambulatory cohort, this apparent flattening cannot be interpreted as a definitive pharmacological plateau, and no formal conclusion regarding a dose–response relationship can be justified (Figure 9). Residual heterogeneity remained entirely absent (I^2^ = 0.00%, τ^2^ = 4.1 × 10^−8^), and the test of residual homogeneity confirmed no unexplained variance between the studies (*p* = 0.912), indicating that the lack of mathematical significance is predominantly a reflection of the small sample size (N = 7) trial arms rather than data inconsistency.

#### 3.2.3. ABPM DBP

**Figure 10:**
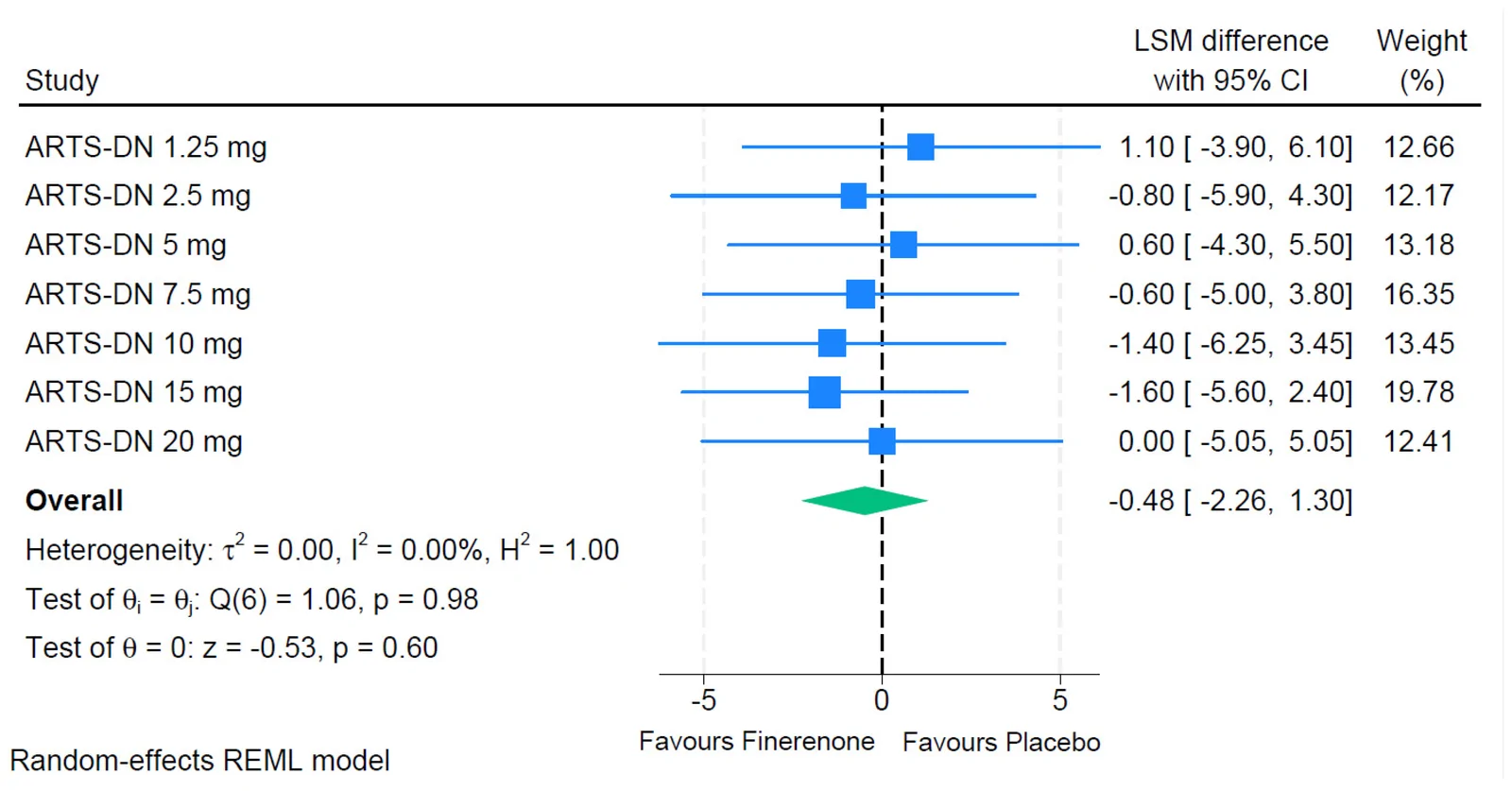
Forest plot assessing the effect of finerenone on ABPM DBP.

To fully characterize the 24-h ambulatory blood pressure profile, a pooled analysis was performed to evaluate the effect of various finerenone doses (ranging from 1.25 mg to 20 mg daily) on ABPM DBP across seven trial arms from the ARTS-DN study. In contrast to the findings observed for systolic ambulatory pressure, the overall pooled analysis demonstrated that finerenone did not induce a statistically significant reduction in ABPM DBP compared with the placebo. The overall pooled LSM difference was −0.48 mmHg, with a 95% CI that widely crossed the line of no effect (−2.26 to 1.30; *p* = 0.60) (Figure 10). The statistical weights were distributed relatively evenly across all evaluated dosage arms, ranging from 12.17% to 19.78%. No clear or significant dose–response pattern was detected among the individual groups. The 1.25 mg arm trended numerically toward the placebo (LSM difference: 1.10 mmHg; 95% CI: −3.90, 6.10), while the 2.5 mg and 5 mg arms reported minimal, non-significant fluctuations of −0.80 mmHg (95% CI: −5.90, 4.30) and 0.60 mmHg (95% CI: −4.30, 5.50), respectively. For the intermediate and high-dose arms (7.5 mg to 20 mg), non-significant numerical reductions were observed up to the 15 mg dose, which achieved the largest absolute numerical drop (LSM difference: −1.60 mmHg; 95% CI: −5.60, 2.40). At the highest dose of 20 mg, the effect size returned to zero (LSM difference: 0.00 mmHg; 95% CI: −5.05, 5.05). None of the individual dosage arms reached statistical significance.

**Figure 11:**
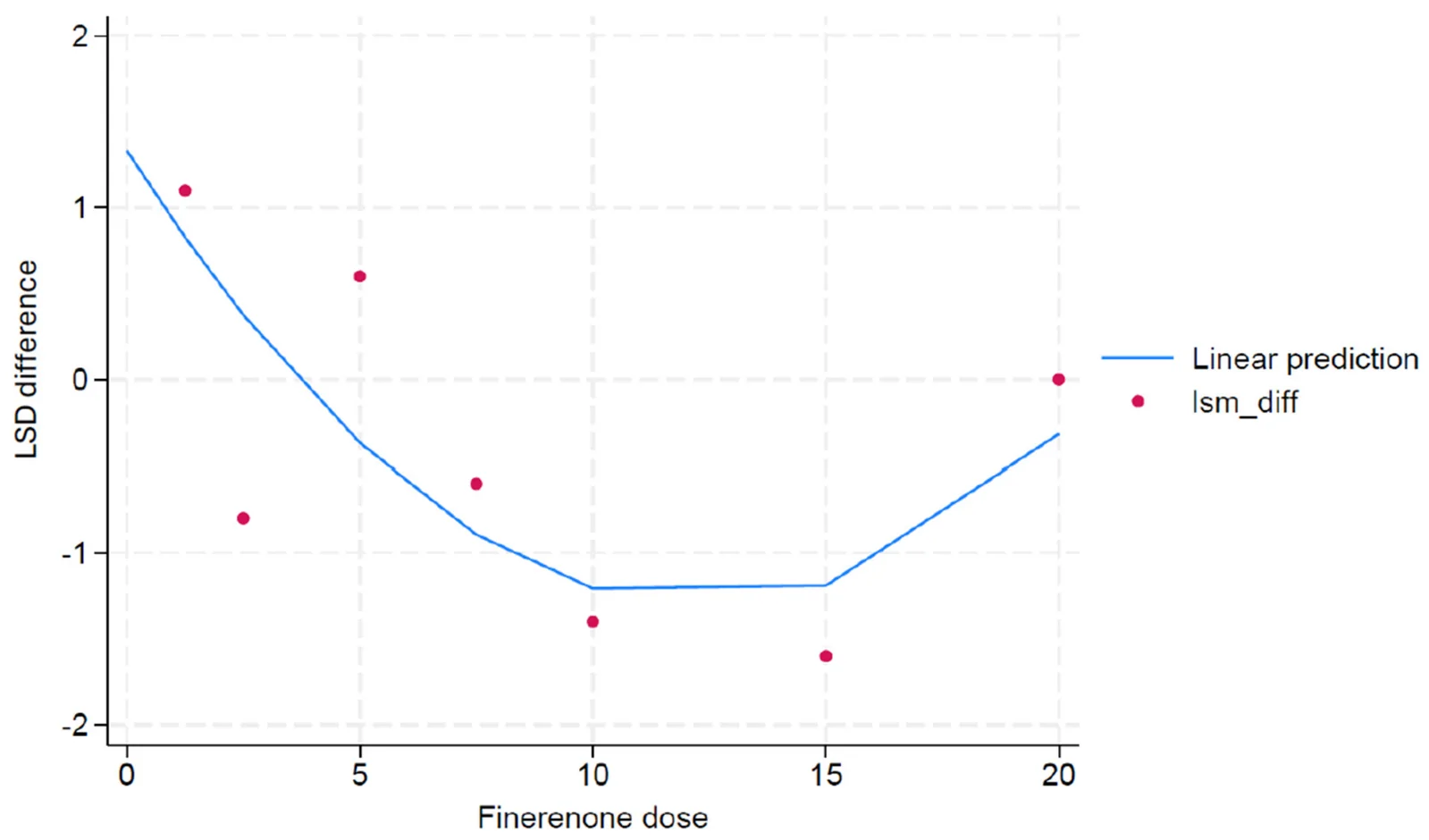
Dose–response effect of finerenone on ABPM DBP.

To mathematically confirm the absence of a linear or monotonic dose–response relationship, a polynomial random-effects meta-regression model was fitted. The model estimated a linear coefficient of −0.426 mmHg per mg increase (95% CI: −1.573 to 0.721; *p* = 0.467) and a quadratic coefficient of 0.017 mmHg (95% CI: −0.036 to 0.071; *p* = 0.530) (Figure 11). Neither the individual predictors nor the omnibus Wald test reached statistical significance (χ^2^ = 0.62, *p* = 0.734), confirming that the variation in finerenone dosage did not exert a mathematically meaningful trend on ABPM DBP. However, visual inspection of the parabolic meta-regression curve reflected a slight, non-significant curvilinear configuration. As illustrated by the polynomial prediction line, the numerical response experienced a modest downward dip at intermediate doses (10 mg and 15 mg), but completely rebounded toward the line of identity at the 20 mg dose, demonstrating a lack of sustained dose-dependent diastolic lowering. Residual heterogeneity remained entirely absent (I^2^ = 0.00%, τ^2^ = 2.7 × 10^−7^), and the test of residual homogeneity confirmed the absence of unexplained inconsistency (*p* = 0.979), reinforcing that the non-significant parabolic trend is likely an artifact of random statistical noise and limited study-level observations (N = 7) rather than a physiological pattern.

## 4. Discussion

This meta-analysis confirms that finerenone induces a modest reduction in systolic blood pressure (SBP), both in-office (−2.83 mmHg) and over 24-h ambulatory monitoring (−7.88 mmHg), with an absence of statistical evidence of heterogeneity (I^2^ = 0.00%). A notable finding is the statistical homogeneity of this hemodynamic effect across diverse patient groups, including CKD-T2DM and heart failure (p_interaction_ = 0.18), indicating a generally uniform numerical direction of the blood pressure reduction. However, this observation must be interpreted with caution; since both the CKD-T1DM and heart failure subgroups are limited to a single clinical trial each, the formal test for subgroup differences lacks the statistical power required to definitively establish true therapeutic consistency across all clinical phenotypes or patient populations.

Our findings are reinforced by recent 2026 data from the FINEARTS-HF trial framework. A longitudinal analysis published in JAMA Cardiology reported a modest placebo-adjusted SBP reduction of ~3.1 mmHg, which closely aligns with our pooled estimate and confirms that finerenone exerts a mild hemodynamic baseline shift rather than acting as a primary antihypertensive drug [19]. Additionally, recent 2026 data from FINEARTS-HF suggested that finerenone safely reduces SBP and fully preserves its long-term cardiovascular benefits even in high-risk patients presenting with apparent treatment-resistant hypertension (aTRH) [20]. However these findings derive from a pre-specified subgroup analysis of a heart failure trial rather than a dedicated randomized trial for resistant hypertension. Therefore, finerenone should be clinically positioned as a drug with modest blood pressure-lowering properties.

The clinical evaluation of these blood pressure changes across different phenotypes is important, particularly in heart failure cohorts where baseline blood pressure levels may be low. In patients with HFmrEF or HFpEF, even a modest SBP reduction could potentially increase the susceptibility to hypotension or orthostatic symptoms. While data from the FINEARTS-HF trial framework suggest that the clinical benefits of finerenone are preserved across these strata, monitoring remains appropriate when introducing the drug into patients with vulnerable hemodynamic baselines.

This hemodynamic profile is further reflected in other hypertensive strata evaluated in smaller cohorts. Gadve et al. [21] reported an office SBP drop (−22 mmHg) in an observational cohort of Indian patients with type 2 diabetes and resistant hypertension, while Li et al. [22] demonstrated daytime SBP reductions (−6.69 mmHg) in primary aldosteronism (PA), including the reversal of suppressed renin levels in 32.7% of patients. When cross-referenced with data from the CONFIDENCE trial [23], a physiological pattern emerged: non-steroidal MR antagonism safely targets hypertensive excursions in high-resistance cohorts (aTRH, resistant hypertension, and PA) [20,21,22,23,24] while avoiding iatrogenic hypotension in normotensive subgroups. These observations are derived from restricted cohorts or subgroup analyses, and dedicated randomized controlled trials are required to draw more definitive clinical conclusions.

Compared with conventional primary antihypertensive agents, such as calcium channel blockers or renin-angiotensin system inhibitors, finerenone features a distinct baseline blood pressure-lowering profile. Its potential added value resides in its mechanism as a non-steroidal MRA, which offers a targeted alternative for patients experiencing MR overactivation without inducing the tolerability limitations typical of steroidal agents.

Crucially, these findings decouple systemic pressure changes from the mechanisms driving long-term organ protection. Causal mediation analyses from the CONFIDENCE trial proved that early SBP reductions accounted for less than 10% of the total urinary albumin-to-creatinine ratio (UACR) lowering, a finding highly concordant with the blood pressure-independent cardiovascular protection observed in FINEARTS-HF. This would suggest that while finerenone might potentially contribute to blood pressure management in resistant hypertension, its clinical efficacy appears to be predominantly mediated by direct, non-hemodynamic tissue-protective, anti-inflammatory, and antifibrotic pathways [24]. Furthermore, finerenone avoided the dose-dependent gonadal side effects traditional to steroidal MRAs, displaying a favorable tolerability profile across the evaluated cohorts [20,21,22,23].

### Limitations

Several limitations warrant consideration. First, the dose–response analysis for 24-h ABPM must be considered strictly exploratory, as it is derived from the varying dosage arms of a single trial (ARTS-DN), which restricts its statistical power and generalizability. Second, meta-regression models were conducted on fewer than 10 studies under zero statistical heterogeneity (I^2^ = 0.00%); thus, these findings are purely exploratory and lack power to confirm the true interaction effects. Third, while our findings regarding 24-h ambulatory SBP showed a numerical reduction of −7.88 mmHg, these data must be interpreted with strict caution. Unlike the primary office SBP analysis that encompasses nearly 20,000 participants across multiple large-scale trials, the ABPM evidence is restricted to a small cohort from the ARTS-DN substudy. Consequently, these wide confidence intervals prevent any definitive conclusions regarding dose–response relationships or definitive plateau thresholds at 15–20 mg, and further large-scale ABPM trials are required. Fourth, strict eligibility criteria, specifically excluding advanced CKD (baseline eGFR <25–30 mL/min/1.73 m^2^), limit applicability to severe cardiorenal cohorts. Finally, the relatively short follow-up in several specialized frameworks precludes definitive conclusions regarding the long-term sustainability of finerenone’s efficacy.

## 5. Conclusions

This meta-analysis establishes that finerenone reduces office SBP by −2.83 mmHg and office DBP by −1.04 mmHg compared with the placebo, with consistency across all clinical cohorts. Furthermore, the agent achieves a reduction in 24-h ambulatory SBP (−7.88 mmHg), suggesting a numerical reduction in SBP that appeared more pronounced at the 15 mg and 20 mg doses than at 10 mg, although this observation is exploratory and limited by the wide confidence intervals of the dose-specific estimates. No statistical divergence was observed for ambulatory DBP. Clinically, finerenone should be positioned as a drug with secondary, modest blood pressure-lowering properties.

## Figures and Tables

**Table 1:** Characteristics of the included trials and the recruited patients.

Trial Population	Finerenone Dose	Population (n)		Gender (Male %)		Age (Mean ± SD or Median (Interquartile))		History of Hypertension (%)		Office SBP at Baseline (mmHg) (Mean ± SD or Median (Interquartile))		Medication Use (%)		Study Duration (Months)
		Finerenone	Placebo	Finerenone	Placebo	Finerenone	Placebo	Finerenone	Placebo	Finerenone	Placebo	Finerenone	Placebo	
**ARTS-DN** [13] CKD-T2DM	From 1.25 mg to 20 mg	1.25 mg = 33 2.5 mg = 22 5 mg = 31 7.5 mg = 34 10 mg = 27 15 mg = 34 20 mg = 31	27	1.25 mg = 87.9 2.5 mg = 86.4 5 mg = 71.0 7.5 mg = 82.4 10 mg = 70.4 15 mg = 76.5 20 mg = 64.5	74.1	1.25 mg = 63.5 ± 8.2 2.5 mg = 63.4 ± 10.6 5 mg = 63.3 ± 8.1 7.5 mg = 63.7 ± 11.5 10 mg = 62.1 ± 7.7 15 mg = 63.8 ± 8.7 20 mg = 66.1 ± 11.3	60.8 ± 8.4	N/A	N/A	1.25 mg = 140.8 ± 14.0 2.5 mg = 134.8 ± 16.6 5 mg = 138.6 ± 17.3 7.5 mg = 140.5 ± 12.4 10 mg = 143.2 ± 12.4 15 mg = 135.8 ± 14.0 20 mg = 138.5 ± 12.2	138.6 ± 13.1	RASi = 100 ACEi = 38.7 ARB = 61.3 BB = 42.5 ABA = 16.0 CCB = 53.8 LD = 20.3 TD = 40.6 CAA = 4.7 N = 3.8	RASi = 100 ACEi = 33.3 ARB = 70.4 BB = 63.0 ABA = 29.6 CCB = 66.7 LD = 18.5 TD = 37.0 CAA = 7.4 N = 0	3
**FIDELIO-DKD** [14] CKD-T2DM	From 10 mg to 20 mg	2833	2841	68.9	71.5	65.4 ± 8.9	65.7 ± 9.2	96.6	97.4	138.1 ± 14.3	138.0 ± 14.4	ACEi = 33.5 ARB = 66.3 D = 55.7 SGLT2i = 4.4 GLP1RA = 6.7	ACEi = 34.9 ARB = 65.0 D = 57.6 SGLT2i = 4.8 GLP1RA = 7.2	31.2
**FIGARO-DKD** [15] CKD-T2DM	From 10 mg to 20 mg	3686	3666	68.6	70.3	64.1 ± 9.7	64.1 ± 10.0	96.1	95.9	135.8 ± 14.0	135.7 ± 14.1	RASi = 99.9 D = 47.4 SGLT2i = 8.5 GLP1RA = 8.4	RASi = 99.9 D = 47.7 SGLT2i = 8.3 GLP1RA = 6.6	10.8
**FINEARTS-HF** [16] HFmrEF-HFpEF	From 10 mg to 40 mg	3003	2998	45.1	45.9	71.9 ± 9.6	72.0 ± 9.7	87.9	89.6	129.5 ± 15.3	129.3 ± 15.3	ACEi = 36.1 ARB = 34.9 ARNI = 8.5 CCB = 31.9 SGLT2i = 13.1 LD = 87.2 TD = 14.3 GLP1RA = 2.6	ACEi = 35.8 ARB = 35.2 ARNI = 8.6 CCB = 33.7 SGLT2i = 14.1 LD = 87.4 TD = 13.4 GLP1RA = 2.9	32
**FINE-ONE** [17] CKD-T1DM	From 10 mg to 20 mg	120	122	65.8	64.8	51.3 ± 14.2	51.9 ± 13.2	86.7	84.4	136.5 ± 15.8	134.2 ± 17.7	ACEi = 49.6 ARB = 50.4 D = 36.1	ACEi = 42.6 ARB = 55.7 D = 36.9	6
**FIVE-STAR** [18] CKD-T2DM	From 10 to 20 mg	51	50	66.7	66.0	73 (65, 80)	73 (63, 77)	92.2	90	130 (122, 146)	129 (118, 141)	ACEi = 7.8 ARB = 56.9 ARNI = 13.7 BB = 29.4 CCB = 66.7 D = 13.7 SGLT2i = 62.7	ACEi = 4.0 ARB = 60.0 ARNI = 16.0 BB = 32.0 CCB = 50.0 D = 14.0 SGLT2i = 66.0	5.5

SD = standard deviation; CKD = chronic kidney disease; T2DM = type 2 diabetes mellitus; HFmrEF = heart failure with mildly reduced ejection fraction; HFpEF = heart failure with preserved ejection fraction; T1DM = type 1 diabetes mellitus; SBP = systolic blood pressure; RASi = renin-angiotensin system inhibitor; ACEi = angiotensin-converting-enzyme inhibitor; ARB = angiotensin-receptor blocker; ARNI = angiotensin receptor-neprilysin inhibitor; BB = beta blocker; ABA = alpha-blocking agents; CCB = calcium-channel blocker; SGLT2i = sodium-glucose cotransporter 2 inhibitor; D = diuretic; LD = loop diuretic; TD = thiazide diuretic; GLP1RA = glucagon-like peptide-1 receptor agonist; CAA = central acting antihypertensives; N = nitrates; N/A = not available.

**Table 2:** Risk of bias assessment of the randomized controlled trials.

	D1	D2	D3	D4	D5	D6	Other Bias
ARTD-DN 2022	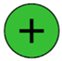	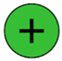	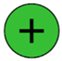	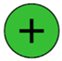	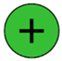	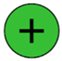	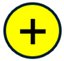
FIDELIO-DKD 2020	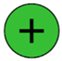	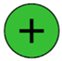	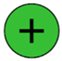	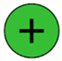	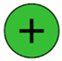	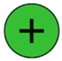	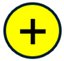
FIGARO-DKD 2021	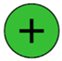	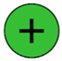	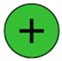	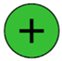	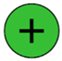	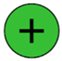	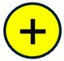
FINEARTS-HF 2024	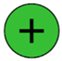	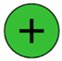	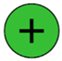	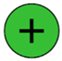	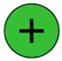	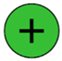	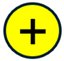
FINE-ONE 2026	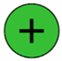	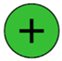	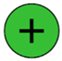	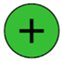	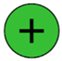	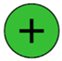	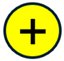
FIVE-STAR 2025	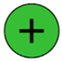	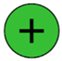	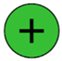	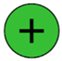	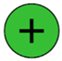	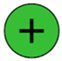	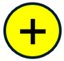

D1: random sequence generation (selection bias). D2: allocation concealment (selection bias) D3: blinding of participants and personnel (performance bias) D4: blinding of outcome assessment (detection bias) D5: incomplete outcome data (attrition bias) D6: selective reporting (reporting bias) Other bias.

## Data Availability

All data are available on reasonable request.

## Supplementary Materials

The following supporting information can be downloaded at: https://www.mdpi.com/article/10.3390/jcm15197694/s1, Figure S1: Search Strategy; Table S1: GRADE evidence table; Table S2: PRISMA checklist.
